# Influence of Cooking Method on the Nutritional Quality of Organic and Conventional Brazilian Vegetables: A Study on Sodium, Potassium, and Carotenoids

**DOI:** 10.3390/foods10081782

**Published:** 2021-07-31

**Authors:** Neide Torres de Castro, Ernandes Rodrigues de Alencar, Renata Puppin Zandonadi, Heesup Han, António Raposo, Antonio Ariza-Montes, Luis Araya-Castillo, Raquel Braz Assunção Botelho

**Affiliations:** 1 Department of Nutrition, University of Brasília, Brasília 70910-900, Brazil; neidetcastro@gmail.com (N.T.d.C.); renatapz@unb.br (R.P.Z.); raquelbotelho@unb.com (R.B.A.B.); 2 Department of Agricultural Engineering, Universidade Federal de Viçosa (UFV), Viçosa 36570-900, Brazil; ernandes.alencar@ufv.br; 3 College of Hospitality and Tourism Management, Sejong University, 98 Gunja-Dong, Gwanjin-Gu, Seoul 143-747, Korea; 4 CBIOS (Research Center for Biosciences and Health Technologies), Universidade Lusófona de Humanidades e Tecnologias, Campo Grande 376, 1749-024 Lisboa, Portugal; 5 Social Matters Research Group, Universidad Loyola Andalucía, C/Escritor Castilla Aguayo, 4, 14004 Córdoba, Spain; ariza@uloyola.es; 6 Facultad de Economía y Negocios, Universidad Andrés Bello, Santiago de Chile 7591538, Chile; luis.araya@unab.cl

**Keywords:** vegetables, organic, cooking method, sodium, potassium, carotenoids

## Abstract

Vegetable consumption is associated with increased health benefits, and vegetables are consumed both in cooked form and raw form in salads. All cooking techniques cause changes in a vegetable’s the nutrient content. Consumers are increasingly health-conscious and have less time to prepare meals, and they do not know which cooking times and cooking methods are best suited to preserve the nutrients. This study aimed to determine the best method of cooking vegetables to maintain minerals (potassium and sodium) and carotenoids. The studied vegetables were broccoli (*Brassica oleracea*, var. Italica), carrots (*Daucus carota*), and zucchini (*Cucurbita moschata*). The cooking methods were: boiling, steaming, combined oven, microwave steaming, and microwave cooking. Samples of organic and conventionally grown vegetables were prepared in triplicate. Samples were analyzed to determine the availability of target minerals and carotenoids in the raw food and in each recommended cooking situation according to technical standards. Only the carrot showed a higher concentration in organic cultivation for carotenoids in raw vegetables, with both zucchini and broccoli having higher concentrations when grown by conventional cultivation. The zucchini from organic cultivation presented a reduction of potassium and sodium, almost consistently, in all cooking techniques. Regarding the conventionally cultivated zucchini, potassium remained stable in boiling. Broccoli from organic and conventional cultivation showed similar potassium levels for boiling and traditional steam cooking. Organic carrots showed easier sodium extraction compared with conventional cultivation. Heat treatment, in general, improves the accessibility of carotenoids.

## 1. Introduction

Adequate vegetable consumption is associated with a healthy diet and reduced risks of noncommunicable diseases [1,2,3,4]. The availability of nutrients in food depends on, among other factors, the cultivation type, pre-processing conditions, and cooking methods [5,6,7].

To better guide individuals regarding vegetable consumption, several studies were performed to analyze the effect of using different cooking methods (i.e., boiling, pressure-cooking, microwave cooking, baking, gridding, frying, and steaming) on the vegetables’ nutrients [1,2,5,7,8,9,10,11,12]. Global differences in nutrient retention between cooking methods can be used to determine which cooking method best favors nutrient retention [12]. Nevertheless, the nutritional value of foods depends on many environmental factors, cultural practices in their preparation, and on the nutritional quality of foods grown by organic farming compared with conventional farming. This is a current topic that has attracted public interest and has generated much discussion. Many consumers choose organic food because they believe it has better health effects. Additionally, consumers believe organic food is safer, more hygienic, and sustainable and that it is without chemical residues and artificial ingredients [13,14,15].

Despite increased health awareness, consumers have less time to prepare meals, and many do not know which cooking times and methods are best suited to preserving the nutrients of the vegetables they consume [10]. Therefore, the question we wanted to answer with this study was whether cooking methods and techniques used in preparing vegetables would induce changes in the availability of water-soluble and fat-soluble nutrients (sodium, potassium, and carotenoids).

## 2. Materials and Methods

This research presents an experimental study with a 2 × 5 × 3 design (2 cultivation methods—conventional and organic); 5 cooking methods (boiling, steam, microwave, steam microwave, and combined oven); with triplicate for each cooking method. The analyzed vegetables were broccoli (*Brassica oleracea*, var. Italica), carrot (*Daucus carota*), and Brazilian zucchini (*Cucurbita moschata*) because they are among the most consumed vegetables in Brazil, according to the latest Family Budget Survey [16]. 

The vegetables were purchased at a local supermarket (Federal District, Brazil) and were stored under refrigeration until they were prepared and cooked. Then, the preparation, including washing and cutting, was carried out according to the procedures described by Miglio et al. [17].

### 2.1. Cooking Methods

The cooking conditions were determined by preliminary experiments [18] conducted for each vegetable. The best cooking times for each method were chosen in a previous study regarding sensorial characteristics [18], and the protocols used followed Pellegrini et al. [19]. Therefore, the cooking methods used were boiling, steam cooking, combined oven, microwave, and steam microwave. Classification tests determined each vegetable’s ideal cooking time and each cooking method by preference, as judged by untrained tasters in a previous study [18]. The methods were classified from the most preferred (1) to the least preferred (4) according to texture, based on a previous study [18]. A section of the ordering method was performed for each vegetable in each cooking method [18].

### 2.2. Sample Analysis

For each cooking time previously determined [18], the samples were prepared in triplicate, adopting the same procedure for the raw vegetables that served as controls. The samples were homogenized and kept frozen in a standard freezer to analyze minerals until their completion and in a freezer at −80 °C to analyze carotenoids until they were lyophilized.

#### 2.2.1. Sodium and Potassium Analysis

The samples were analyzed in triplicate to determine the sodium and potassium availability in the raw vegetable and in each of the recommended cooking situations, according to technical standards of the Adolfo Lutz Institute [20].

Therefore, 5 g of each sample was weighed in a porcelain capsule. The samples were carbonized in an electric plate and subsequently incinerated in a muffle furnace at 550 °C. After cooling, 30 mL of hot distilled water was added, and the contents were stirred with a glass stick. The solution was transferred to a 100 mL volumetric flask with the aid of a funnel. The capsule, the glass stick, and the funnel were washed with two more portions of 30 mL of hot water, and then all the liquid was filtered and transferred to another volumetric flask. After being completely cold, the flasks were completed with the necessary amount of distilled water to reach 100 mL and were agitated before directly reading at the flame photometer. In cases where a higher concentration of the mineral was found than the equipment’s original reading capacity, the solution was diluted in a proportion of 1:5.

The photometer was calibrated with the standard solutions for each of the minerals, following the concentrations established for the equipment. The readings were carried out one at a time by inserting the photometer catheter directly into the balloon or transferring part of its contents to a beaker when necessary. Based on the reading result, a calculation was performed to find the concentration in mg/100 g of each raw or cooked fresh vegetable.

#### 2.2.2. Carotenoids Analysis

The extraction of carotenoids was carried out according to Rodriguez-Amaya [21], with adaptations, and all stages were developed in an environment with controlled temperature and low light. Lyophilized samples (0.5 g or 1 g) were macerated in a mortar, using 20 mL of refrigerated acetone (10 °C), and homogenized for one minute. Each weighed sample depended on the concentration of carotenoids in the vegetable according to food composition tables. The extract was vacuum filtered through a Büchner funnel with filter paper, collecting the filtrate in a 250 mL conical vacuum flask. The extraction was repeated until the residue was white. In the end, the mortar, residue, and funnel were washed with acetone, collecting everything in the conical vacuum flask. Acetone was used for the initial extraction of carotenoids from the samples. In a 250 mL separating funnel, 20 mL of petroleum ether was placed. The extract was carefully transferred to the funnel, washing the conical vacuum flask with acetone and transferring the extract to the funnel. An amount of 300 mL of distilled water was carefully added through the funnel walls to avoid the formation of an emulsion.

For the total removal of acetone and transfer of carotenoids to petroleum ether, washings were carried out with distilled water. After separating the phases, the lower aqueous phase was discarded, washing three more times with 150 mL of distilled water to remove all acetone. The lower phase was discarded entirely in the last wash, so as not to lose part of the upper phase. The funnel outlet was dried with paper towels to prevent small amounts of water from mixing with the ethereal phase. The ethereal phase was collected in a 25 mL volumetric flask covered with aluminum foil, passing the solution through a glass funnel containing 7.55 g of anhydrous sodium sulfate to remove residual water, using glass wool in the funnel to hold the sodium sulfate. The funnel was washed with the petroleum ether collected in the volumetric flask. The volume of the volumetric flask was completed with petroleum ether. The absorbance was read on a spectrophotometer at a wavelength ranging from 380 to 680 nm. The following equation was used to calculate the carotenoid content.

Total carotenoid concentration (Ct):

(1) $$\mathrm{Ct}\;(\mu g/g)=\frac{A\;\times \;V\;\times 10^{4}}{E^{1\%}{}_{1\mathrm{cm}}\times m}$$

A = absorbance at absorption peak

V = final sample volume (mL)

m = sample mass (g)

E^1%^_1cm_ = extinction coefficient − β-carotene = 2592 in petroleum ether [22] or 2500 when the reading is not in that range of the spectrum [23].

### 2.3. Data Analysis

The raw food data were used as a control sample to compare changes that occurred during processing. The analysis aimed to perform the quantitative measurement of the changes that occurred; the percentages of alteration of nutrients in processed foods were analyzed on the raw sample of the same food. The verification followed the same premise regarding carotenoids—comparing the percentage variation in content in raw and cooked samples based on the different cooking methods.

The results for sodium, potassium, and carotenoids were submitted to analysis of variance (ANOVA), and means were compared by Fisher’s test (*p* < 0.05) using XLstat software, version 2017.6 (Addinsoft, France, 2017).

## 3. Results

Carotenoids

Table 1 shows the changes in each vegetable’s carotenoid content in each cooking method for each cultivation experiment, considering the raw vegetable as the unit against which all changes were evaluated.

For zucchini, in addition to steaming in the conventional cultivation experiment, all other cooking methods presented more carotenoids than the raw sample. For broccoli, the same was observed for the organic cultivation experiment with more carotenoids for all the vegetables in the different cooking methods. This tendency was not observed for broccoli from the conventional cultivation process. Carrots presented a different pattern from zucchini and broccoli. All cooking methods presented fewer carotenoids in carrots than the raw vegetable for the organic experiment. For conventional cultivation, only cooking in a combined oven presented higher levels of carotenoids from carrots.

Table 2 shows the potassium and sodium content changes in each vegetable in each cooking method for each cultivation experiment, considering the raw vegetable as the unit against which all changes were evaluated.

In Table 2, it is possible to observe that all cooking methods present less potassium and sodium for organic zucchini. For the conventional cultivation experiment, microwave cooking presented higher potassium and sodium levels, as did microwave steaming for sodium. For organic broccoli, microwave cooking presented higher levels of potassium and sodium. However, potassium was in higher levels for the conventional broccoli when cooking in microwave steaming, as was sodium when cooking in the combined oven. For carrots, the results were different for zucchini and broccoli. For organic carrots, sodium was higher in all of the cooking methods when compared with the raw vegetable. Whereas for potassium, steaming, microwave, and microwave steaming presented the highest levels. For conventional carrots, potassium as well as sodium were higher in combined oven cooking. Microwave cooking carrots also presented higher sodium levels.

## 4. Discussion

### 4.1. Carotenoids

#### 4.1.1. Zucchini and Broccoli

Zucchini and broccoli have carotenoids stored in chloroplasts, which are quite sensitive to abiotic events [24]. Therefore, there may have been a difference in the release of their products (carotenoids) in different types of cultivation, considering that they were not cultivated in the same period [25]. However, it is also possible that there was an influence of the cultivation system, considering the characteristics of nutrition and moisture inherent to the specificity of organic cultivation [26], to which the chloroplast is especially sensitive.

In the case of zucchini, as it is a green (immature) vegetable, it still has carotenoids stored in chloroplasts. Some fruits with green mesocarps, such as kiwi (*Actinidia* spp. Delicious), keep their green color and typical chloroplasts, similar to leaves, until maturity and complete harvest. Zucchini also has a lower concentration of carotenoids, increasing as the fruit/vegetable ripens, and the chloroplasts are transformed into chromoplasts [27]. Since high levels of total carotenoids are directly related to a higher density of chloroplasts, and therefore to dark green foods, it was not expected to find large concentrations of carotenoids in zucchini [27]. Carotenoid content was similar between organic and conventional cultivation.

In the organic cultivation of zucchini, the most significant extraction was obtained with vegetables cooked in dry heat (microwave), which seems to be a natural consequence of the concentration of water loss and which did not positively impact the tissues facilitating the process. However, the same did not happen with the zucchini of conventional cultivation. Despite the apparent difference between the quantities extracted in the different cooking methods, there was no statistically significant difference. For organic broccoli, the carotenoid concentration was higher for steam and microwave steam cooking. There is a difference in the amount of fibers and proteins found in this vegetable. According to the Brazilian food composition table [28], broccoli has about 91% water, while zucchini about 96%; broccoli has 2.9 g of fiber in 100 g of raw vegetables, while zucchini has 1.2 g/100 g; concerning protein, zucchini has 0.6 g/100 g compared with 3.6 g/100 g in broccoli. For broccoli, the results suggest that the degradation of protein complexes and the softening of fibers were more critical to the increase in the extraction of carotenoids than the concentration obtained with water reduction in the dry heat process—due to the different composition between the two vegetables.

Ryan et al. [29], evaluating in vitro bioaccessibility of conventional cultivation vegetables, found that the zucchini, when submitted to dry heat cooking in the microwave, grilled, boiled, or steamed, had reduced bioaccessibility for carotenoids compared with the raw vegetable. Results were different from our findings, which showed more significant amounts for boiling, microwaving, and microwave steaming. However, all tested cooking processes (boiling, steam, microwave, and grill) improved the micellization capacity of carotenoids when subjected to the in vitro digestion process, highlighting the importance of heat treatment for improving the absorption of these nutrients [29].

In other studies, the broccoli obtained from conventional agriculture [30] and organic [31] cooked-in-water (boiling), steamed, and steam microwaved and at maximum power presented values different from ours, with easier extraction of carotenoids in vegetables of conventional cultivation than in the organic ones. However, the boiling cooking time was shorter: just 5 min. The steam cooking was carried out for 20 min but with the vegetables already placed before the steam production, which occurred after 8 min. Therefore, data are difficult to compare because of different cooking times, latitudes, and longitudes, aspects that directly influence the production of carotenoids.

The study used as a model for this research [19] presented results quite different for conventionally grown broccoli. For comparison, we calculated their cooked/raw ratio. It was 0.7 for boiling, 0.6 for microwave, 1.0 for traditional steam, and 1.2 for the combined oven. Thus, only the combined oven showed a similar value compared with the conventionally grown broccoli in our research. However, it is important to consider that we do not know the time of cultivation, but we do know that latitude and longitude directly influence carotenoid production.

In a study measuring β-carotene and lutein [32] in broccoli, the authors found that the extraction of both increased after steaming and boiling. The cooking water was collected and analyzed, but no carotenoids were found in this water, showing no carotenoid loss in the cooking process. Any difference between raw and treated samples is due to the tissues’ structure changes, which become difficult for extraction. A similar result (lutein and β-carotene) was found in a study performed with boil-cooking, steaming in the microwave, and traditional steaming [33]. The authors showed that the longer the cooking time, the greater the amount of carotenoids extracted. Steam and microwaves had better results than boiling, and they concluded that easier extraction could translate into greater bioavailability of the nutrient when ingested [33]. Steaming and microwave steaming were also the best cooking methods for organic broccoli in our study, as presented in Table 1.

Miglio et al. [17] analyzed the total carotenoids in zucchini (*Cucurbita pepo*), broccoli (*Brassica oleracea*), and carrots (*Daucus Carota*) from conventional cultivation submitted to boiling and combined oven. For zucchini, there was a reduction of carotenoids in the two cooking methods. For broccoli, there was an increase in the carotenoids extraction for the two cooking methods. Unfortunately, cooking times were not reported, but as part of a larger study group, it is assumed that they were the same as those used in the study of Pellegrini et al. [19]: 8 min for boiling and 13 min for microwave steam for broccoli. For broccoli, the results were very similar to those found in our study. However, concerning zucchini, the values were much lower, probably due to the different cultivar, which may impact tissue type with a different fiber composition. Therefore, the behavior of carotenoids in these tissues is consistently changing.

For broccoli, in both types of cultivation, the lowest extraction occurred when using the microwave. It suggests that this organic vegetable had less water (greater dry matter), as having less water in a vegetable tends to occur with this type of cultivation. Therefore, broccoli benefited from cooking—hydrating its fibers and facilitating the extraction of carotenoids. Even though microwave cooking presented lesser extraction than other cooking methods, cooking presented better carotenoids’ extraction than raw broccoli, showing the importance of the procedure. For zucchini, cooking was also important for higher levels of carotenoid extraction.

#### 4.1.2. Carrot

When comparing raw carrots and carrots cooked in different methods, raw organic carrots presented higher levels; however, cooking in a combined oven brought higher carotenoid extraction for conventional carrots. All other cooking methods were equal to the raw carrot. This pattern was different from zucchini and broccoli.

For Rodriguez-Amaya et al. [34], some factors make the analysis of carotenoids in food very difficult. These factors are the variation in the amount of carotenoids among samples, the nature of the food matrices, the existence of many carotenoids, and the non-uniform distribution of carotenoids between samples.

Carrot showed worse accessibility when cooked in a combined oven (50% compared with raw carrots) and better in a microwave oven (80% compared with the control), possibly due to the natural concentration of moisture loss. For carrots of conventional cultivation, the extraction of carotenoids, in general, was superior to that found in organic cultivation. However, except for the combined oven (30% increase) and microwave dry heat (loss of 30%), there were no differences between them, and the values were similar to those of raw vegetables.

Based on the results of a study with carrots boil-cooked with added salt [35], the ratio between the values found for the raw and cooked carrots was 0.9, indicating a reduction in the accessibility of the total carotenoids in this cooking process. With the addition of curry or coconut shavings, accessibility had not changed significantly [35]. The addition of these components points to the fact that variations in conventional preparations can modify the availability of carotenoids.

Nunn et al. [36], analyzing broccoli and carrots in steamed cooking and steamed cooking in the microwave, found higher values for carrots and slightly lower for conventionally grown broccoli, with similar results for both methods. Mazzeo et al. [37] analyzed bleached and frozen carrots and then thawed them in a combined oven (20 min) and by boiling (12 min). The relationship between the values found in their work for raw and cooked carrots was 0.9 for both boiling and combined oven. Thus, results were quite different from ours, where the combined oven presented the best extraction. However, it is important to consider that we do not know the time of cultivation or whether the latitude and longitude of cultivation are different.

A study [38] showed the accessibility of 0.8 of β-carotene (the only carotenoid evaluated) for carrots boiled for 10 min. This result was similar to ours for conventional cultivation. Pressure cooking was also evaluated, and for both cooking techniques, spices (Curcuma longa, tamarind, and powdered onion) and citric acid were added [38]. The retention was slightly changed and differed by cooking methods and based on the added spices. The addition of citric acid and onion reduced the availability of β-carotene in boiling and increased it in pressure cooking, which points to other factors that may enhance the accessibility of carotenoids.

Miglio et al. [17] found that the amount of carotenoids extracted was reduced when the carrot was cooked in a combined oven (0.9), and in the case of boil-cooking, there was a slight increase in accessibility (1.1)—different results from ours in which the combined oven showed the best conditions for increasing the accessibility of carotenoids (1.3) in carrots of conventional cultivation. It is essential to mention that the fiber content of the carrot is much higher than that of the other analyzed vegetables in this study: 3.2 g/100 g, and therefore, there is expected to be greater difficulty in extracting nutrients from carrots, considering its extra dose of protection.

An analysis of the cellular structure of the carrot using electron microscopy [39] showed that it has cells with cell walls with fibrous cellulose microfibrils embedded in a matrix of polysaccharides, hemicellulose, and pectin. The cells are small in size, paired, with thick and denser walls with pectin content that can reduce the porosity of the cell wall. A study [40] on carrot and potato using confocal microscopy to evaluate the spatial arrangement of cells and their geometric parameters (such as the size, shape, and orientation of cells within the tissue) concluded that the fracture of tissues composed of larger cells requires less work (a lower concentration of cell walls, in the tissue, leads to increased failure). Thus, it seems clear that for the release of the carotenoid content of the carrot tissues, there must be a significant increase in temperature for a more extended period to supply the energy necessary for the rupture large quantities of the dense cell walls, in addition to the energy necessary for the dissolution of pectin.

Carotene crystals appear to be the primary storage substructure in the chromoplast of carrots and are among the most difficult to solubilize during digestion. A study showed that in carrots, there is a medium difficulty in extracting carotenoids [41]. The authors worked with homogenized and raw carrots, where the raw carrot had a significantly higher extraction of carotenoids concerning the treatments used [41] and not differing from our results.

Lemmens et al. [42], in the analysis of carrots cut into pieces of about 1 cm^3^ cooked by boiling for 3 and 25 min and also raw and fractionated in 12 different particle sizes (<40 μm, 41–80 μm, 81–125 μm, 126–160 μm, 161–250 μm, 251–500 μm, 501–800 μm, 801–1000 μm, 1001–1400 μm, 1401–2000 μm, 2001–4000 μm, and 4001–6300 μm), found that the release of β-carotene from the carrot tissue depends on the particle size, even indicating that less intense heat treatment—3 min—in particles smaller than 125 μm, showed a pronounced increase in the extraction of carotenoids compared with the raw samples and those cooked longer (25 min). This study is important, pointing out that it is not only the cooking method and time that influence carotenoids extraction.

### 4.2. Sodium and Potassium

For zucchini cultivated conventionally, the two processes that least involved water (microwave dry heat and microwave steam) were the ones that presented higher amounts of sodium and potassium, as presented in Table 2. However, in organic cultivation, all cooking methods showed very similar values, which were lower than those of the raw zucchini. For broccoli, the behavior of sodium and potassium was similar in the organic cultivation method. Potassium increased in cooking by microwave and also in traditional steaming. For sodium, there was only an increase in microwave cooking. In conventional cultivation, potassium levels were similar to raw only in traditional steam cooking, and microwave steaming broccoli presented higher levels than raw broccoli. As for sodium, only combined oven cooking presented higher levels than the raw sample. For carrots, sodium was increased in all cooking methods for organic cultivation, while for conventional cultivation, only the boiling method showed a reduction. There was also an increase in potassium concentration, which was lower only for the combined oven in organic cultivation and for microwave steaming for conventional cultivation.

Considering that sodium is an eminently extracellular ion, it is expected that it will be more easily leached in any submitted treatment that involves water. Therefore, it might present values lower than those of raw vegetables. However, with the loosening of the cell walls, the quantities of sodium existing in the intracellular environment would also be released, resulting in expectations that the sodium values would subsequently increase [43,44]. For dry heat processes, regardless of the amount of moisture in the vegetable, there should be an increase in this nutrient’s concentration. Regarding the differentiation by type of cultivation, considering that potassium and sodium sometimes act together or in counter-regulation, potassium is expected to be higher in conventionally grown vegetables grown using NPK as a fertilizer. Consequently, sodium would follow potassium [45].

Potassium is an eminently intracellular ion found in smaller amounts in the extracellular medium. It naturally depends on the loosening of the cell wall to be released to the external environment. Thus, it would be expected that the dosed values would always be a little higher when the food was subjected to thermal treatments, especially in processes that involve less water, as this would favor its leaching since it is water-soluble [43,44]. An increase in potassium and sodium is expected when vegetables are subjected to cooking processes that do not involve their leaching into the cooking water.

In a study [46] intending to reduce potassium in broccoli, carrots, and potatoes, the traditional boiling technique and microwave reduced potassium (compared with raw) for both carrots and broccoli, resulting in greater potassium loss by microwave boiling compared with traditional boiling. However, broccoli had a more significant potassium loss when cooked in a pressure cooker [46].

A study [47] in which potassium and sodium content in organic and conventional carrot juice were evaluated found that although some differences in potassium content were detected, they were not statistically significant. A review [48] compared 32 organic/conventional equivalent pairs and concluded that the potassium level in samples of conventional plants was higher in 58% of the pairs, which would be expected by the type of fertilizer used. The cultivation method did not influence the sodium content in the carrot. However, the author reports that older studies (before the year 2000) showed lower sodium levels and higher potassium concentrations in organic vegetables, similar to our results.

A meticulous and interesting study with the use of microwave cooking in humid heat was carried out using three different powers (1000, 750, and 500 W) with two cooking times each (2.5 and 5 min) and with the addition of different amounts of water (100 and 150 mL) for 150 g of broccoli from conventional cultivation. Nutrients were measured in the samples and the cooking water [49]. Regarding potassium, there was minimal variation between cooking times and between cooked and raw vegetables, regardless of the power and amount of water. However, the quantity of the mineral retained in the food found in the cooking water was greater than that found in the raw vegetable. In some cases, there was no mineral loss in the cooking water (longer cooking times), suggesting that part of the intracellular potassium was extracted. For sodium, the results were already a little different. Despite the similarity of the results between the times, potency, and quantities extracted and leached in the cooking water, it is interesting to note that in all of them, the sum of the two portions (food and cooking water) was lower than that the amount found in raw food, suggesting an ion exchange (Na +/K +) between the intracellular and extracellular media when the cell wall is damaged.

This study presents some limitations regarding the different cultivation periods of organic and conventional samples. Although the three vegetables were cultivated in the same city, the regions within the city were different for both types of cultivation. Additionally, the ripening stage of each vegetable was not evaluated.

## 5. Conclusions

Carrot showed a higher concentration of carotenoids in organic cultivation, with both zucchini and broccoli having higher concentrations in conventional cultivation. Zucchini and carrots showed higher potassium concentrations in organic cultivation, while the opposite was found with broccoli. All vegetables of conventional cultivation had a higher sodium content than organic cultivation. The zucchini of organic cultivation presented a reduction of potassium and sodium, almost consistently, in all cooking techniques. For conventionally cultivated zucchini, potassium did not change in boiling and had a 40% loss in the combined oven. On the other hand, there was a 10% increase in the potassium extraction for traditional steaming and microwave steaming, and a 20% increase in the microwave dry heat due to the water loss in this cooking method. The reduction in sodium content was 20% for boiling and combined oven, 10% for traditional steaming, and an increase of 40% and 50% for microwave in dry heat and steam, respectively.

Broccoli from organic and conventional cultivation showed similar results to potassium for boiling and traditional steam. There was no change in potassium levels for organically cultivated broccoli that was cooked in microwave steam, and there was an increase of 20% in conventionally cultivated broccoli. In the combined oven, it was quite similar, with a 30% loss for conventional broccoli cultivation and a 20% loss for organic cultivation. In comparison, there was a 60% increase in organic cultivation in microwave dry heat and a 20% loss in conventional cultivation. For potassium from carrots, the organic and conventional cultivation behavior was quite similar, being the same for boiling and traditional steam. Organic carrots showed easier sodium extraction than conventional cultivation. The greatest extraction was obtained by microwave steaming—50%. Heat treatment, in general, improves the accessibility of carotenoids and improves carotenoid accessibility to a greater extent in organic vegetables than in conventionally grown vegetables, except for carrots, where accessibility has been significantly reduced in vegetables grown by organic cultivation. In conventional cultivation, there were slight carotenoid reductions or maintenance of carotenoid levels, without statistical significance.

## Figures and Tables

**Table 1:** Changes in the content of carotenoids in organic and conventional cultivation vegetables according to the cooking method.

Cooking Method	Organic Cultivation		Conventional Cultivation	
	Carotenoid Content µg/g	Cooked/Raw	Carotenoid Content µg/g	Cooked/Raw
Zucchini				
Raw	22.9 ± 2.1 ^d^	1.0	37.6 ± 4.0 ^b^	1.0
Boiling (10 min)	32.1 ± 1.9 ^c^	1.4	49.6 ± 4.7 ^a^	1.3
Steaming (12 min)	40.0 ± 4.2 ^b^	1.7	38.1 ± 4.7 ^b^	1.0
Microwave steaming (12 min)	29.7 ± 2.0 ^c^	1.3	54.6 ± 4.4 ^a^	1.5
Microwave (16 min)	47.6 ± 2.6 ^a^	2.1	48.6 ± 2.3 ^a^	1.3
Combined oven (13 min)	37.3 ± 2.8 ^b^	1.6	48.4 ± 5.0 ^a^	1.3
Broccoli				
Raw	54.5 ± 3.7 ^d^	1.0	77.4 ± 5.8 ^ab^	1.0
Boiling (8 min)	138.5 ± 12.2 ^b^	2.5	85.0 ± 0.7 ^a^	1.1
Steaming (10 min)	200.2 ± 21.1 ^a^	3.7	81.5 ± 10.7 ^a^	1.1
Microwave steaming (10 min)	185.4 ± 2.9 ^a^	3.4	77.8 ± 1.8 ^ab^	1.0
Microwave (8 min)	88.3 ± 1.0 ^c^	1.6	62.8 ± 6.6 ^b^	0.8
Combined oven (15 min)	151.5 ± 11.1 ^b^	2.8	90.9 ± 16.3 ^a^	1.2
Carrot				
Raw	554.9 ± 44.6 ^a^	1.0	472.3 ± 29.5 ^b^	1.0
Boiling (10 min)	350.4 ± 20.6 ^c^	0.6	412.8 ± 34.5 ^b^	0.9
Steaming (10 min)	320.2 ± 31.8 ^cd^	0.6	401.9 ± 12.2 ^b^	0.9
Microwave steaming (10 min)	320.7 ± 9.2 ^cd^	0.6	455.9 ± 43.1 ^b^	1.0
Microwave (10 min)	443.0 ± 13.0 ^b^	0.8	316.7 ± 61.1 ^c^	0.7
Combined oven (11 min)	298.7 ± 19.2 ^d^	0.5	627.6 ± 51.3 ^a^	1.3

^a–d^ For each vegetable and attribute, in columns, the values followed by the same letters do not differ by the Fischer test (*p* > 0.05). Carotenoid content is expressed based on the vegetable’s fresh weight.

**Table 2:** Changes in potassium and sodium content in vegetables from organic and conventional cultivation, according to cooking method.

Cooking Method	Organic Cultivation				Conventional Cultivation			
	Potassium		Sodium		Potassium		Sodium	
	mg/100 g	Cooked/Raw	mg/100 g	Cooked/Raw	mg/100 g	Cooked/Raw	mg/100 g	Cooked/Raw
Zucchini								
Raw	1.513.3 ± 71.3 ^a^	1.0	1.5 ± 0.1 ^a^	1.0	150.4 ± 4.5 ^bc^	1.0	6.3 ± 0.6 ^bc^	1.0
Boiling (10 min)	1.217.4 ± 32.1 ^b^	0.8	1.2 ± 0.1 ^b^	0.8	152.4 ± 3.0 ^bc^	1.0	4.7 ± 0.4 ^d^	0.8
Steaming (12 min)	1.090.9 ± 56.7 ^c^	0.7	0.8 ± 0.0 ^c^	0.5	162.5 ± 11.5 ^ab^	1.1	5.4 ± 0.5 ^cd^	0.9
Microwave steaming (12 min)	1.090.0 ± 23.0 ^c^	0.7	1.2 ± 0.1 ^b^	0.8	148.9 ± 3.1 ^c^	1.1	9.7 ± 0.5 ^a^	1.5
Microwave (16 min)	1.069.1 ± 22.7 ^c^	0.7	1.1 ± 0.1 ^b^	0.7	172.2 ± 10.0 ^a^	1.2	8.8 ± 0.5 ^a^	1.4
Combined oven (13 min)	1.035.5 ± 8.9 ^c^	0.7	1.2 ± 0.3 ^b^	0.9	86.9 ± 3.0 ^d^	0.6	7.3 ± 1.1 ^b^	0.8
Broccoli								
Raw	81.4 ± 3.0 ^b^	1.0	7.8 ± 0.3 ^b^	1.0	159.4 ± 4.2 ^bc^	1.00	9.4 ± 0.5 ^b^	1.0
Boiling (8 min)	55.6 ± 4.7 ^c^	0.7	7.9 ± 0.1 ^b^	1.0	107.6 ± 4.1 ^e^	0.7	6.3 ± 0.5 ^c^	0.7
Steaming (10 min)	79.7 ± 2.6 ^b^	1.0	5.9 ± 0.0 ^c^	0.8	154.3 ± 14.9 ^c^	1.0	9.6 ± 1.0 ^b^	1.0
Microwave steaming (10 min)	77.8 ± 4.0 ^b^	1.0	5.8 ± 0.3 ^c^	0.7	184.0 ± 9.4 ^a^	1.2	9.1 ± 0.4 ^b^	1.0
Microwave (8 min)	132.9 ± 6.2 ^a^	1.6	9.8 ± 0.7 ^a^	1.2	176.0 ± 14.6 ^ab^	1.1	7.0 ± 0.0 ^c^	0.7
Combined oven (15 min)	61.4 ± 4.9 ^c^	0.7	8.2 ± 0.1 ^b^	1.0	133.41.2 ^d^	0.8	11.6 ± 0.1 ^a^	1.2
Carrot								
Raw	1.155.9 ± 46.7 ^de^	1.0	3.6 ± 0.1 ^d^	1.0	169.7 ± 5.9 ^b^	1.0	7.4 ± 0.5 ^c^	1.0
Boiling (10 min)	1.108.4 ± 90.7 ^e^	1.0	4.7 ± 0.4 ^b^	1.3	167.5 ± 4.7 ^b^	1.0	6.5 ± 0.6 ^d^	0.9
Steaming (10 min)	1.352.6 ± 37.3 ^ab^	1.1	4.4 ± 0.3 ^bc^	1.2	181.4 ± 7.6 ^b^	1.1	7.2 ± 0.3 ^cd^	1.0
Microwave steaming (10 min)	1.324.1 ± 30.5 ^bc^	1.1	5.2 ± 0.2 ^a^	1.4	129.0 ± 11.8 ^c^	0.8	7.6 ± 0.4 ^bc^	1.0
Microwave (10 min)	1.434.4 ± 24.6 ^a^	1.2	4.2 ± 0.2 ^c^	1.1	173.6 ± 16.5 ^b^	1.0	8.4 ± 0.6 ^ab^	1.1
Combined oven (11 min)	1.247.7 ± 56.5 ^cd^	0.54	5.3 ± 0.2 ^a^	1.5	205.8 ± 1.4 ^a^	1.2	9.2 ± 0.5 ^a^	1.2

^a–d^ For each vegetable and attribute, in columns, the same letter values do not differ by the Fischer test (*p* > 0.05). Potassium and sodium contents are expressed based on the vegetable´s fresh weight.

## Data Availability

This study did not report any data.

## References

[1] Guillén S., Mir-Bel J., Oria R., Salvador M.L. (2016). Influence of cooking conditions on organoleptic and health-related properties of artichokes, green beans, broccoli and carrots. Food Chem..

[2] Gili R.V., Leeson S., Montes-Chañi E.M., Xutuc D., Contreras-Guillén I.A., Guerrero-Flores G.N., Martins M.C.T., Pacheco F.J., Pacheco S.O.S. (2019). Healthy vegan lifestyle habits among argentinian vegetarians and non-vegetarians. Nutrients.

[3] Lynch H., Johnston C., Wharton C. (2018). Plant-Based Diets: Considerations for Environmental Impact, Protein Quality, and Exercise Performance. Nutrients.

[4] Hartmann Y., Botelho R.B.A., Akutsu R.D.C.C.d.A., Puppin Zandonadi R. (2018). Consumption of Fruits and Vegetables by Low-Income Brazilian Undergraduate Students: A Cross-Sectional Study. Nutrients.

[5] Fabbri A.D.T., Crosby G.A. (2016). A review of the impact of preparation and cooking on the nutritional quality of vegetables and legumes. Int. J. Gastron. Food Sci..

[6] Bongoni R., Verkerk R., Dekker M., Steenbekkers L.P. (2014). Consumer behaviour towards vegetables: A study on domestic processing of broccoli and carrots by Dutch households. J. Hum. Nutr. Diet..

[7] Kala A., Prakash J. (2006). The comparative evaluation of the nutrient composition and sensory attributes of four vegetables cooked by different methods. Int. J. Food Sci. Technol..

[8] Poelman A.A.M., Delahunty C.M. (2011). The effect of preparation method and typicality of colour on children’s acceptance for vegetables. Food Qual. Prefer..

[9] Kaloustian J., Alhanout K., Amiot-Carlin M.J., Lairon D., Portugal H., Nicolay A. (2008). Effect of water cooking on free phytosterol levels in beans and vegetables. Food Chem..

[10] Zhou B., Yamanaka-Okumura H., Seki S., Tatano H., Adachi C., Takeda E. (2014). What influences appetite more: Eating approaches or cooking methods?. J. Med. Invest..

[11] Kumar S., Aalbersberg B. (2006). Nutrient retention in foods after earth-oven cooking compared to other forms of domestic cooking. J. Food Compos. Anal..

[12] Bernhardt S., Schlich E. (2006). Impact of different cooking methods on food quality: Retention of lipophilic vitamins in fresh and frozen vegetables. J. Food Eng..

[13] Zhao X., Chambers E., Matta Z., Loughin T.M., Carey E.E. (2007). Consumer sensory analysis of organically and conventionally grown vegetables. J. Food Sci..

[14] Gąstoł M., Domagała-Świątkiewicz I., Krośniak M. (2011). Organic versus conventional—A comparative study on quality and nutritional value of fruit and vegetable juices. Biol. Agric. Hortic..

[15] Pearson D., Henryks J., Jones H. (2010). Organic food: What we know (and do not know) about consumers. Renew. Agric. Food Syst..

[16] IBGE (2011). Pesquisa de Orçamentos Familiares: Análise do Consumo Alimentar Pessoal no Brasil.

[17] Miglio C., Chiavaro E., Visconti A., Fogliano V., Pellegrini N. (2008). Effects of different cooking methods on nutritional and physicochemical characteristics of selected vegetables. J. Agric. Food Chem..

[18] Torres de Castro N., de Lacerda L., Rodrigues de Alencar E., Assunção Botelho R.B. (2020). Is there a best technique to cook vegetables?—A study about physical and sensory aspects to stimulate their consumption. Int. J. Gastron. Food Sci..

[19] Pellegrini N., Chiavaro E., Gardana C., Mazzeo T., Contino D., Gallo M., Riso P., Fogliano V., Porrini M. (2010). Effect of Different Cooking Methods on Color, Phytochemical Concentration, and Antioxidant Capacity of Raw and Frozen Brassica Vegetables. J. Agric. Food Chem..

[20] Instituto Adolfo Lutz (2008). Métodos Físico-Químicos Para Análise de Alimentos.

[21] Rodriguez-Amaya D.B. (2001). A Guide to Carotenoid Analysis in Foods.

[22] Telfer A., Pascal A., Gall A., Britton G., Liaaen-Jensen S., Pfander H. (2008). Chapter 14. Carotenoids in: Photosynthesis. Carotenoids Volume 4: Natural Functions.

[23] Britton G., Springer (1997). Carotenoids. Natural Food Colorants.

[24] Pogson B.J., Ganguly D., Albrecht-borth V. (2015). Insights into chloroplast biogenesis and development. BBA Bioenerg..

[25] Britton G., Khachik F., Britton G., Liaaen-Jensen S., Pfander H. (2009). Chapter 3. Carotenoids in Food. Carotenoids Volume 5: Nutrition and Health.

[26] Orsini F., Maggio A., Rouphael Y., De Pascale S. (2016). “Physiological quality” of organically grown vegetables. Sci. Hortic..

[27] Schweiggert R.M., Carle R. (2017). Carotenoid deposition in plant and animal foods and its impact on bioavailability. Crit. Rev. Food Sci. Nutr..

[28] Núcleo de Estudos e Pesquisas em Alimentação (NEPA), Universidade Estadual de Campinas (UNICAMP) Tabela Brasileira de Composição de Alimentos. https://www.cfn.org.br/wp-content/uploads/2017/03/taco_4_edicao_ampliada_e_revisada.pdf.

[29] Ryan L., O’Connell O., O’Sullivan L., Aherne S.A., O’Brien N.M. (2008). Micellarisation of carotenoids from raw and cooked vegetables. Plant Foods Hum. Nutr..

[30] Dos Reis L.C.R., De Oliveira V.R., Hagen M.E.K., Jablonski A.A., Flôres S.H., De Oliveira Rios A. (2015). Carotenoids, flavonoids, chlorophylls, phenolic compounds and antioxidant activity in fresh and cooked broccoli (Brassica oleracea var. Avenger) and cauliflower (Brassica oleracea var. Alphina F1). LWT Food Sci. Technol..

[31] Dos Reis L.C.R., De Oliveira V.R., Hagen M.E.K., Jablonski A.A., Flôres S.H., De Oliveira Rios A. (2014). Effect of cooking on the concentration of bioactive compounds in broccoli (Brassica oleracea var. Avenger) and cauliflower (Brassica oleracea var. Alphina F1) grown in an organic system. Food Chem..

[32] Gliszczyńska-Swigło A., Ciska E., Pawlak-Lemańska K., Chmielewski J., Borkowski T., Tyrakowska B. (2006). Changes in the content of health-promoting compounds and antioxidant activity of broccoli after domestic processing. Food Addit. Contam..

[33] Hwang E.-S., Kim G.-H. (2012). Effects of various heating methods on glucosinolate, carotenoid and tocopherol concentrations in broccoli. Int. J. Food Sci. Nutr..

[34] Rodriguesz-Amaya D.B., Kimura M., Amaya-Farfan J. (2008). Fontes Brasileiras de Carotenóides. Tabela Brasileira de Composição de Carotenoides em Alimentos.

[35] Priyadarshani A.M.B., Chandrika U.G. (2007). Content and in-vitro accessibility of pro-vitamin A carotenoids from Sri Lankan cooked non-leafy vegetables and their estimated contribution to vitamin A requirement. Int. J. Food Sci. Nutr..

[36] Nunn M.D., Giraud D.W., Parkhurst A.M., Hamouz F.L., Driskell J.A. (2006). Effects of cooking methods on sensory qualities and carotenoid retention in selected vegetables. J. Food Qual..

[37] Mazzeo T., N’Dri D., Chiavaro E., Visconti A., Fogliano V., Pellegrini N. (2011). Effect of two cooking procedures on phytochemical compounds, total antioxidant capacity and colour of selected frozen vegetables. Food Chem..

[38] Gayathri G.N., Platel K., Prakash J., Srinivasan K. (2004). Influence of antioxidant spices on the retention of β-carotene in vegetables during domestic cooking processes. Food Chem..

[39] Jeffery J., Holzenburg A., King S. (2012). Physical barriers to carotenoid bioaccessibility. Ultrastructure survey of chromoplast and cell wall morphology in nine carotenoid-containing fruits and vegetables. J. Sci. Food Agric..

[40] Zdunek A., Umeda M. (2005). Influence of cell size and cell wall volume fraction on failure properties of potato and carrot tissue. J. Texture Stud..

[41] Jeffery J.L., Turner N.D., King S.R. (2012). Carotenoid bioaccessibility from nine raw carotenoid-storing fruits and vegetables using an in vitro model. J. Sci. Food Agric..

[42] Lemmens L., Van Buggenhout S., Van Loey A.M., Hendrickx M.E. (2010). Particle size reduction leading to cell wall rupture is more important for the ??-carotene bioaccessibility of raw compared to thermally processed carrots. J. Agric. Food Chem..

[43] Adams C.E., Erdman J.W., Karmas E., Harris R.S. (1988). Effects of Home Food Preparation Practices on Nutrient Content of Foods. Nutritional Evaluation of Food Processing.

[44] Miller D.D., Srinivasan D., Kirk L., Parkin O.R.F. (2010). Minerais. Quimica de Alimentos de Fennema.

[45] Reeve J.R., Hoagland L.A., Villalba J.J., Carr P.M., Atucha A., Cambardella C., Davis D.R., Delate K. (2016). Organic Farming, Soil Health, and food Quality: Considering Possible Links.

[46] Copetti C., de Oliveira V.R., Kirinus P. (2010). Avaliação da redução de potássio em hortaliças submetidas a diferentes métodos de cocção para possível utilização na dietoterapia renal. Rev. Nutr..

[47] Domagała-Świątkiewic I., Gąstoł M. (2012). Comparative study on mineral content of organic and conventional carrot, celery and red beet juices. Acta Sci. Pol. Hortorum Cultus.

[48] Benbrook C., Zhao X., Yáñez J. (2008). New evidence confirms the nutritional superiority of plant-based organic foods. State Sci. Rev. Nutr. Super. Org. Foods.

[49] López-Berenguer C., Carvajal M., Moreno D.A., Garcia-Viguera C. (2007). Effects of microwave cooking conditions on bioactive compounds present in broccoli inflorescences. J. Agric. Food Chem..

